# Trans Fats in Spanish Pastries and Their Influence on Mesenchymal Stem Cell Behavior In Vitro and Related Health Risks

**DOI:** 10.3390/foods14132247

**Published:** 2025-06-25

**Authors:** Camilo Zamora-Ledezma, José Manuel Martínez-Hernandez, Jeevithan Elango, Judit Garcia-Garrido, Juana María Morillas-Ruiz, Eliana Díaz-Cruces, Pablo Javier Miró-Colmenárez, Ezequiel Zamora-Ledezma

**Affiliations:** 1 Bioengineering & Regenerative Medicine Research Group (Bio-ReM), Escuela de Ingeniería, Arquitectura y Diseño (EIAD), Universidad Alfonso X el Sabio (UAX), Avenida de la Universidad 1, Villanueva de la Cañada, 28691 Madrid, Spain; 2 Green and Innovative Technologies for Food, Environment and Bioengineering Research Group (FEnBeT), Faculty of Pharmacy and Nutrition, Universidad Católica San Antonio de Murcia (UCAM), Campus de Los Jerónimos 135, Guadalupe, 30107 Murcia, Spain; 3 Faculty of Pharmacy and Nutrition, Universidad Católica San Antonio de Murcia (UCAM), Campus de Los Jerónimos 135, Guadalupe, 30107 Murcia, Spain; jmmartinez3@ucam.edu (J.M.M.-H.); jmmorillas@ucam.edu (J.M.M.-R.); 4 Department of Biomaterials Engineering, Faculty of Health Sciences, Universidad Católica San Antonio de Murcia (UCAM), Campus de Los Jerónimos 135, Guadalupe, 30107 Murcia, Spain; jelango@ucam.edu; 5 HiTech, Sport & Health Innovation Hub, Universidad Católica San Antonio de Murcia (UCAM), Campus de Los Jerónimos 135, Guadalupe, 30107 Murcia, Spain; jgarcia66@ucam.edu; 6 Law Ecotechnology and Innovation Keys for the 21st Century Development Research Group, Faculty of Law, Universidad Católica San Antonio de Murcia (UCAM), Campus de Los Jerónimos 135, Guadalupe, 30107 Murcia, Spain; eliana.cruces10@gmail.com (E.D.-C.); pjmiro@ucam.edu (P.J.M.-C.); 7 Facultad de Ciencias Jurídicas, Universidad a Distancia de Madrid (UDIMA), Collado Villalba, 28400 Madrid, Spain; 8 Laboratorio Funcionamiento de Agroecosistemas y Cambio Climático FAGROCLIM, Departamento de Ciencias Agrícola, Facultad de Ingeniería Agrícola, Universidad Técnica de Manabí, Lodana 13132, Ecuador; ezequiel.zamora@utm.edu.ec

**Keywords:** health risks, regulations, trans fatty acids (TFAs), mesenchymal stem cells (MSCs), Spanish pastry products, cardiovascular health risks, food regulatory compliance

## Abstract

Trans fats are linked to numerous chronic diseases and cellular dysfunction; however, Spain has not implemented effective regulatory measures to restrict their presence in food products. This study addressed these gaps by analyzing trans fat content in commercial pastries sold in Spain and their biological impacts on mesenchymal stem cells, further examining its compliance with international guidelines. Also, a novel and scalable method for extracting fatty acids from pastry samples was developed and applied, enabling precise analysis using gas chromatography alongside sensory property assessments. The findings revealed significant variability in TFA levels across samples. To assess the biological implications of these TFAs, mesenchymal stem cells (MSCs) were cultured to perform dose–response experiments using two selected pastry samples with the highest TFA content. Cellular adhesion, cytotoxicity, and proliferation were evaluated through MTT assays, bright-field, and fluorescence staining using FITC and DAPI markers. Results demonstrated dose-dependent impacts of TFAs on MSC viability, including reduced adhesion and proliferation alongside increased cytotoxicity. This study underlines the need for stricter regulatory frameworks to monitor TFA levels worldwide, including in Spain’s food industry. Additionally, it highlights the potential health risks associated with excessive TFA consumption, particularly concerning cellular health and growth mechanisms, which provide insights into its potential bioaccumulation implications. These findings provide a foundation for further research into dietary guidelines and industrial practices aimed at minimizing TFA exposure while promoting public health safety.

## 1. Introduction

The presence of trans fats in the foods we consume daily invites us to reflect on how closely our collective choices align with current scientific understanding [1,2,3]. Despite the well-documented negative health effects of trans fatty acids (TFAs), regulatory measures in many countries, including, but not limited to, Spain, remain insufficient to effectively curb their use in the food industry [3,4]. This ongoing gap between evidence and policy highlights the need for stronger action to safeguard public health and underscores the importance of advancing toward more responsible food regulations grounded in scientific research. Trans fatty acids, commonly known as trans fats, are a type of unsaturated fat that can be found in both natural and industrially processed foods. Their consumption has been associated with a range of adverse health outcomes, including, in particular, an increased risk of cardiovascular disease [5,6,7].

Recent in vitro studies have provided mechanistic insights into the cellular toxicity of food-derived trans fatty acids (TFAs). For example, Hirata et al. demonstrated that industrial TFAs, such as elaidic and linoelaidic acid, promote DNA damage-induced apoptosis in various mammalian cell types by enhancing mitochondrial reactive oxygen species (ROS) generation and activating the JNK signaling pathway, thus amplifying cell death responses [8]. Similarly, they showed that TFAs can act as enhancers of inflammation and apoptosis in cell models, particularly when cells are exposed to additional stressors, highlighting the unique pro-apoptotic role of TFAs compared to their cis isomers and saturated fatty acids [8]. All of these findings highlight the importance of considering cellular-level effects when evaluating the health risks associated with dietary TFAs [9]. Indeed, they induce cellular damage in mesenchymal stem cells (MSCs) through three primary pathways: (1) mitochondrial apoptosis via ROS overproduction, activating JNK-p38 MAPK cascades and caspase-dependent pathways; (2) inflammatory amplification through enhanced TNF-α/TNFR1 signaling and ASK1-p38 activation; and (3) metabolic disruption involving impaired fatty acid oxidation, ER stress, and caspase-3-mediated apoptosis [10]

Fatty acids are esterified hydrocarbon chains classified by two primary structural features: (1) carbon chain length (C12:0 to C24:0) and (2) degree of unsaturation (saturated, monounsaturated, or polyunsaturated). Shorter-chain fatty acids (C12:0–C16:0) are metabolized rapidly via β-oxidation, serving as immediate energy substrates, whereas longer chains (C18:0–C24:0) are preferentially incorporated into lipoproteins or stored in adipose tissue, contributing to atherogenic potential when oxidized. Saturated fatty acids (SFAs), lacking double bonds (e.g., C16:0 palmitic acid), exhibit linear conformations that enhance membrane rigidity and promote low-density lipoprotein (LDL) cholesterol (also commonly known as bad cholesterol) synthesis via hepatic upregulation of HMG-CoA reductase. Conversely, unsaturated fatty acids, including monounsaturated (MUFAs, e.g., C18:1 oleic acid) and polyunsaturated (PUFAs, e.g., C18:2 linoleic acid), introduce kinks from cis-configured double bonds, reducing lipid packing density and improving fluidity, thereby modulating cardiovascular risk [11,12,13,14,15,16]. The health risks of fatty acids are therefore associated with both structure and dietary context [3,13,17]. For instance, SFAs with 12–16 carbons (lauric C12:0, myristic C14:0, palmitic C16:0) demonstrate strong associations with elevated low-density lipoprotein (LDL) and cardiovascular disease (CVD), as evidenced by meta-analyses linking a 5% energy increase in SFAs to a 17% rise in CVD mortality [2,5,18]. For their part, trans fatty acids (TFAs), such as trans-C18:1 (elaidic acid), formed during industrial hydrogenation are particularly harmful. As a matter of fact, their linear geometry mimics SFAs but with amplified LDL elevation and high-density lipoprotein (HDL) suppression, doubling coronary risk per 2% energy intake [5,19,20]. Notably, even naturally occurring TFAs (e.g., in ruminant fats) exhibit similar toxicity. In contrast, PUFAs like omega-3 α-linolenic acid (C18:3) and omega-6 linoleic acid (C18:2) are cardioprotective at balanced ratios (1:4–1:10), but they also attenuate inflammation and endothelial dysfunction. However, as reported widely in the literature, excessive omega-6 intake may propagate oxidative stress if antioxidant defenses are inadequate [4]. Also, long-chain SFAs (C20:0–C24:0) and very-long-chain MUFAs (C22:1 erucic acid) are less studied but implicated in ectopic lipid deposition and myocardial lipidosis, respectively [6,13,14,21].

As far as their impact on health, the World Health Organization (WHO) recommends that intake of trans fatty acids (TFAs) should not exceed 1% of total daily energy intake, which is equivalent to less than 2.2 g per day for an adult based on a 2000 kcal diet. Additionally, both the WHO and European legislation set a maximum limit of 2 g of TFA per 100 g of fat in food products [22]. In Spain, the regulation in force since 2021 adopts this same standard, and various studies have shown that most products available on the market comply with this requirement, reflecting a downward trend in TFA content in processed foods [5,23,24]. However, despite these recommendations and regulations [25,26], there are significant challenges in the supervision and control of TFA both globally and in Spain. Currently, there are no institutions exclusively dedicated to monitoring the TFA content in foods. Supervision largely depends on self-regulation by the food industry and occasional inspections by health authorities, which limits the ability to detect systematic non-compliance or changes in product formulations. According to the WHO, only 43% of the world’s population is protected by policies that restrict TFA. Furthermore, in Spain, there is no independent system that continuously verifies compliance with the established limits [27]. Another important challenge is that the current regulatory approach focuses on setting a maximum TFA limit per 100 g of fat in the product but does not consider key aspects, such as frequency of consumption or the body’s capacity to handle intake. For example, although a food may comply with the legal limit of 2 g of TFA per 100 g of fat [5], frequent or large-quantity consumption can lead to a daily intake much higher than that recommended by the WHO. Thus, if a person consumes 1 kg of a food containing the maximum permitted limit, they would be ingesting 20 g of TFA, which is nine times the recommended limit. This regulatory gap is especially relevant in contexts where the diet is rich in ultra-processed foods or in populations with eating habits that favor repeated consumption of products containing TFA [23,24].

Based on these challenges, this study is structured around three central pillars. First, the trans fat (TFA) content in 11 of the most widely consumed commercial pastry samples in Spain was quantitatively analyzed using gas chromatography and an improved, scalable method for fatty acid extraction. Second, it was determined whether the detected TFA levels comply with international guidelines (<2% of total fat). Third, the biological effects of these fats on mesenchymal stem cells (MSCs) were examined through viability (MTT), cell adhesion, proliferation, and cytotoxicity assays. These biological experiments were complemented by bright-field microscopy and staining techniques (H&E, FITC, DAPI), allowing us to obtain detailed structural information. This complete approach enables simultaneous observation of cellular architecture and precise localization of labeled molecules, providing a complete understanding of cellular responses to TFAs.

## 2. Materials and Methods

### 2.1. Samples’ Description

The details of the product names and codes used throughout the analysis are summarized in Table 1.

### 2.2. Fat Extraction Through Ultrasound

**Figure 1:**
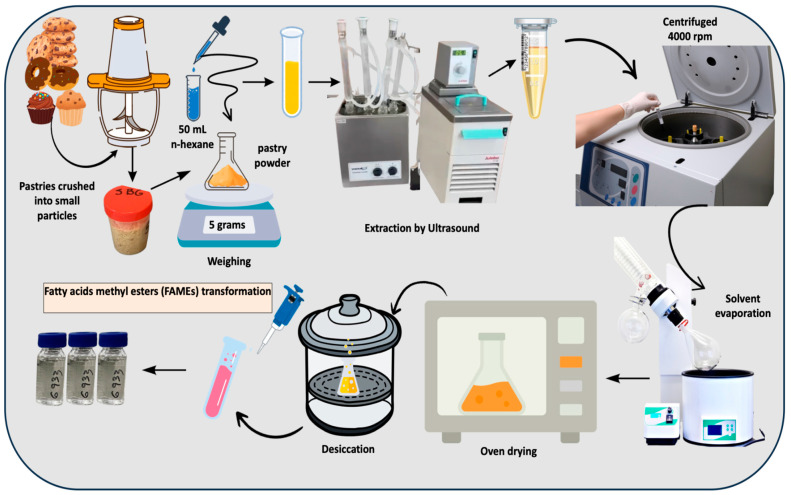
Schematic representation of fat extraction through ultrasonication.

Fat was extracted from the bakery samples (including different types of cookies, cakes, and sponge cakes) using an ultrasound-assisted method revisited to ensure efficient recovery of lipids [28]. The procedure used for fat extraction is depicted in Figure 1. Prior to extraction, the bakery samples were chopped and/or ground into small particles to increase the surface area and improve solvent penetration during the fat extraction process [29]. Then, approximately 5 g was weighed into a pre-dried flask. This step was repeated in triplicate for each product to ensure accuracy and reproducibility. Next, 50 mL of n-hexane were added to each flask, and the mixture was allowed to stand covered for one hour to promote interaction between the solvent and the sample. The flasks were then placed in an ultrasonic bath set at 25 °C for 30 min, with a coolant system attached and maintained at 10 °C to prevent solvent loss due to evaporation. After sonication, the mixtures were transferred to 50 mL conical tubes and centrifuged at 4000 rpm for 5 min to separate the liquid fat extract from the solid residue. The clear supernatant was collected and transferred to a round-bottom flask. Furthermore, the hexane from the previous mixture was removed using a rotary evaporator set at 40 °C and 60 rpm. To ensure all traces of solvent were eliminated, the extracts were further dried in an oven at 40 °C and then placed in a desiccator until they reached room temperature and a constant weight. The total fat content was calculated by weighing the residue, allowing for precise determination of the fat yield in each sample.

### 2.3. Fatty Acid Methyl Ester Preparation and Characterization Through Gas Chromatography-Mass Spectrometry (GC-MS)

Before GC-MS analysis, the extracted fatty acids were converted into methyl esters (FAMEs) to ensure compatibility with gas chromatography. With this aim, 100 μL of the extracted oil from each sample was used for fatty acid analysis [30]. The oil was mixed with 4 mL of hexane and vortexed for one minute to ensure complete dissolution. Then, 0.5 mL of a 2 M potassium hydroxide solution in methanol was added, and the mixture was vortexed again for five minutes to convert the fatty acids into their methyl esters (FAMEs) [31]. The mixture was centrifuged at 3000 rpm for 5 min, and 2 mL of the upper (hexane) layer containing the FAMEs was collected and diluted to 5 mL with hexane. These prepared samples were stored in 1.5 mL amber glass vials until analysis. The fatty acid composition was determined using gas chromatography–mass spectrometry (GC-MS) following the ISO 12966-1:2014 standard [31]. Chromatographic analysis was performed using an Agilent HP 8890 system equipped with an HP-88 cyanopropyl silica capillary column (60 m × 0.25 mm × 0.2 µm) employing hydrogen as the carrier gas in Split mode (1:50 ratio) [31]. The FAMEs were injected into the GC-MS system equipped with a capillary column specifically designed for fatty acid separation. The column’s temperature was programmed to gradually increase, allowing for optimal separation of the different fatty acids, including the critical trans fatty acid isomers. Optimized thermal conditions included an initial isothermal phase at 172 °C for 10 min, followed by a 1.5 °C/min ramp to 210 °C and a final hold for 4.67 min, completing the analysis in 40 min with effective resolution of cis/trans isomers and fatty acids from C12:0 to C24:0. The separated compounds were identified by comparing their mass spectra and retention times with those of certified reference standards and spectral libraries. Quantification was achieved using calibration curves prepared from known standards. A commercially available FAME standard mixture (Supelco 37 Component FAME Mix, Sigma-Aldrich, St. Louis, MO, USA), covering the expected range of fatty acids, was used for calibration and identification, following ISO 12966-2:2017 and AOCS Ce 1h-05 guidelines [31]. The results were expressed as the percentage of each fatty acid relative to the total fatty acids in the sample [32]. Special attention was given to the quantification of industrial trans fatty acids (TFAs), such as trans-oleic acid (trans-C18:1) and trans-linoleic/linolenic acids (trans-C18:2 and trans-C18:3), due to their known adverse health effects. Indeed, compound identification relied on retention times compared to certified standards, while quantification used percentage area normalization with response factor corrections. Trans fatty acids were quantified with expanded uncertainty calculated at 95% (k = 2) per protocol. Method validation included paired sample analyses demonstrating <2% variations in major components, confirming the robustness of both the chromatographic system and the derivatization protocol. The measured TFA levels were compared with the World Health Organization (WHO) recommendations, which advise keeping TFA intake below 1% of total daily energy to reduce health risks [33].

### 2.4. Determination of Fatty Acid Quality and Composition Indices

To determine the quality and composition indices of the extracted fatty acids, standard analytical methods were applied according to international normative protocols (ISO and AOAC).

#### 2.4.1. Acid Value

The acid value was measured by titrating a known quantity of fat sample dissolved in a previously neutralized mixture of diethyl ether and ethanol with a standardized alcoholic solution of potassium hydroxide (KOH), using phenolphthalein as an indicator. Results were expressed in milligram of KOH per gram of fat (AOAC 940.28; ISO 660:2020) [34,35].

#### 2.4.2. Anisidine Value

The anisidine value, which evaluates secondary oxidation products, such as aldehydes and ketones, was determined through reaction with p-anisidine reagent, measuring absorbance at 350 nm according to ISO 6885:2016 and the AOCS method Cd 18–90 [36].

#### 2.4.3. Peroxide Value

The peroxide value, indicative of primary oxidation levels, was determined by reacting the fat with potassium iodide (KI) and subsequently titrating the liberated iodine with sodium thiosulfate (Na_2_S_2_O_3_). Results were reported as milliequivalents (meq) of O_2_ per kilogram of fat, in accordance with ISO 3960:2017 and AOAC 965.33 [37,38].

#### 2.4.4. Iodine Value

The iodine value, reflecting the degree of unsaturation in the fat, was determined using the Wijs method through reaction with an iodine–bromide solution (IBr), followed by titration of excess reagent with sodium thiosulfate. Results were expressed as grams of iodine absorbed per 100 grams of fat (AOAC 920.158; ISO 3961:2018) [39,40].

#### 2.4.5. Saponification Value

The saponification value was obtained by saponifying the sample with an excess of alcoholic KOH solution and subsequently titrating the residual alkali with standardized hydrochloric acid (HCl). Results were expressed in milligram of KOH per gram of fat (AOAC 920.160; ISO 3657:2020). All measurements were performed at least in triplicate to ensure the accuracy and reproducibility of the obtained result [41,42].

### 2.5. Raman and FTIR Vibrational Spectroscopy Characterization

For the vibrational spectroscopic characterization of samples, both Fourier Transform Infrared (FTIR) and Raman spectroscopy were employed to provide complementary insights into the molecular structure and the composition. Samples were prepared following standard laboratory protocols to ensure reproducibility, handled exclusively with powder-free gloves, and stored in airtight containers to avoid contamination and moisture uptake. FTIR spectra were collected using a Jasco FT/IR-6X1typeA spectrometer equipped with an ATR PRO ONE X accessory at a 45° incident angle, employing a standard light source and a TGS detector. Each measurement consisted of 40 accumulations at a resolution of 2 cm^−1^, with zero filling enabled and cosine apodization applied to optimize spectral quality. The aperture and scan speed were set to automatic (5 mm and 2 mm/s, respectively), and a filter frequency of 12,800 Hz was used. Spectral data were acquired over a range of 4000 to 400 cm^−1^ with a data interval of 0.48 cm^−1^, yielding 8000 data points per spectrum, and they were recorded as percent transmittance (%T) versus wavenumber. Raman spectra were obtained using a Jasco PR-1w spectrometer operating with a 785 nm laser at medium power, with an exposure time of 1 s per accumulation and 40 accumulations per sample to enhance the signal-to-noise ratio. The Raman spectral range covered 200 to 3000 cm^−1^ at a data interval of 1 cm^−1^, resulting in 3000 data points per spectrum, and measurements were recorded as intensity (arbitrary units) versus Raman shift. Both FTIR and Raman instruments were calibrated using standard reference materials prior to measurement, and background correction was performed using the manufacturer’s standard procedures. All measurements were conducted at ambient temperature, and each sample was analyzed in triplicate to ensure reproducibility and analytical rigor. Spectral interpretation focused on identifying the number and position of absorption and scattering bands, with particular attention to the single bond region (2500–4000 cm^−1^), carbonyl and aromatic regions (1700–1600 cm^−1^), and the fingerprint region (600–1500 cm^−1^). Functional group assignments were made by comparing observed peaks to literature values and reference spectra, and all spectral assignments were cross-validated using published databases and correlation tables. The combined use of FTIR and Raman spectroscopy provided robust qualitative and quantitative information about the chemical composition, structure, and potential degradation products present in the analyzed materials.

### 2.6. Cell Culture

Human bone mesenchymal stem cells (hMSCs) (ATCC PCS-500-012, Order Ref. No. 86605340) were obtained from LGC Standards, Barcelona, Spain. Primary hMSC cultures (5 × 10^5^ cells/vial) were established following the supplier’s guidelines. Mesenchymal stem cells (MSCs) were selected as the cellular model for this study due to their high physiological relevance in regenerative processes and their sensitivity to metabolic and oxidative stress. MSCs are considered as relevant as cardiomyocytes or neurons for evaluating the cytotoxic effects of dietary trans fatty acids given their multipotency and involvement in systemic tissue responses. Typically, cells were maintained in mesenchymal-specific medium (PCS-500-030) supplemented with 5% fetal bovine serum (FBS) (Gibco, Waltham, MA, USA), 1% antibiotic mix (penicillin–streptomycin–amphotericin B) (PCS-999-002), and growth factors (15 ng/mL rh IGF-1, 125 pg/mL rh FGF-b, 2.4 mM L-Alanyl-L-Glutamine; PCS-500-041). Cultures were incubated for 5–7 days with medium replacement twice per week. Upon reaching 80% confluence, cells were sub-cultured through trypsinization with 0.25% trypsin-EDTA, followed by centrifugation at 200× *g* for 5 min. The resulting cell pellet was resuspended in MSC culture medium and utilized in subsequent experiments. Cells from passages 3–7 were employed for all assays [43,44].

#### 2.6.1. Cytotoxic Effect of TFA

To assess the cytotoxic effect, TFA samples were prepared by dissolving them in 1% DMSO at various concentrations. Mesenchymal stem cells (MSCs) at a density of 1.5 × 10^4^ were plated in 96-well plates and treated with TFA concentrations of 0.001%, 0.005%, 0.01%, 0.05%, 0.1%, 0.5%, and 1% over periods of 1, 2, and 3 days. Two controls were included in all experiments: (i) untreated cells (no additives) and (ii) cells treated with 1% DMSO (vehicle control) without TFA. At each time point, the cells were treated with MTT reagent (0.5 mg/mL in PBS) for 3 h, followed by washing with PBS. Subsequently, 100 µL of DMSO was added, and the mixture was incubated for 15 min. The optical density was then measured at 570 nm using a plate reader. All cytotoxicity and microscopy experiments (including cell morphology/adhesion assessments) were performed in triplicate.

#### 2.6.2. Morphology of MSCs with TFA

To examine cell morphology, mesenchymal stem cells (MSCs) at a density of 5 × 10^4^ were plated in 24-well plates and subjected to treatment with TFA samples, as previously described. The cells were treated with TFA for durations of 1, 2, and 3 days. Following treatment, they were rinsed with PBS, fixed using a solution of 4% paraformaldehyde and 2.5% glutaraldehyde, permeabilized with 0.1% Triton X-100, and stained with FITC and DAPI for one hour each in the dark. Subsequently, the cells were washed with PBS, and images were obtained using a fluorescence microscope. In parallel, bright-field images of cells treated with TFA samples were captured following the above procedure without FITC and DAPI staining using light microscopy.

### 2.7. Statistical Analysis

All data were processed using GraphPad Prism 10 (GraphPad Software Inc., San Diego, CA, USA). To assess statistical significance among groups, a one-way analysis of variance (ANOVA) was performed. Results with a *p*-value less than 0.05 were considered statistically significant.

## 3. Results

### 3.1. Fatty Acids’ Characterization

**Figure 2:**
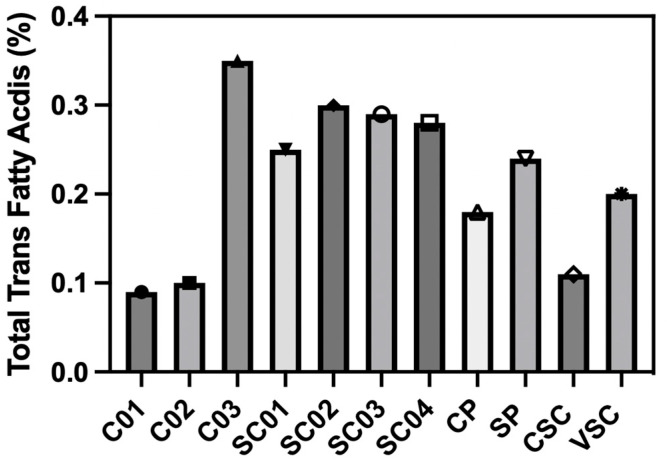
Total trans fatty acid content (%) in fat extracts from 11 traditional Spanish pastries. Each bar represents the mean value for a specific pastry sample, highlighting the variation in trans fat content across different products.

The fatty acid profiles of eleven Spanish pastry samples (C01-C03, SC01-SC04, CP, SP, CSC, and VSC) reveal significant variations in their composition specifically in both total fat content and fatty acid profiles across products, as determined by fatty acid characterization through gas chromatography–mass spectrometry (GC-MS). The fatty acid composition and trans fatty acid content of each sample are presented in Table 2. Total fat ranged from 1.81% in Sponge Cake-04 (SC04) to 34.4% in Chocolate Palmier (CP), with other high-fat products including Sponge Cake Pastry (SP, 27.9%), Chocolate Sponge Cake (CSC, 25.6%), and Valenciana Sponge Cake (VSC, 25.3%). For its part, the oleic acid (C18:1) was one of the most abundant fatty acids in Cookie-01 (C01, 77.80%) and Cookie-02 (C02, 75.60%), suggesting notable use of olive oil or other high-oleic sources, while palmitic acid (C16:0) was predominant in Cookie-03 (C03, 39.89%) and CP (28.91%), indicating the likely presence of palm oil or similar fats. On the other hand, linoleic acid (C18:2) was highest in VSC (55.04%) and SP (52.03%), reflecting the use of polyunsaturated-rich vegetable oils in these sponge cakes. It is worth mentioning that all samples contained trans fatty acids (TFAs) below the 2% international regulatory threshold, with the highest values observed in C03 (0.35%), Sponge Cake-02 (SC02, 0.30%), and Sponge Cake-03 (SC03, 0.29%). Furthermore, the total trans fatty acid content in the fat extracts from the 11 Spanish pastries is plotted in Figure 2. The results show a clear variation in trans fat levels among the different pastry samples, with all values remaining below 0.4% [24,45,46,47].

Table 3 presents the quality and composition indices of four selected Spanish pastries (Chocolate Palmier, Cocoa cake, Sobao, and Valencian cupcakes), which were chosen from the larger group of 11 pastries initially analyzed for trans fatty acid content. This reduction in sample sizes was implemented to have analytical depth for different types of pastries, as determination of multiple quality parameters (acidity, anisidine, peroxide, iodine, and saponification values) requires significantly more resources compared to standard trans fatty acid screening through GC-MS. The selection of these four pastries was based on their representation of diverse trans fatty acid levels (from relatively low to moderate) as well as their widespread consumption in Spain, ensuring the relevance of this study to common dietary exposures. Among the four pastries analyzed, the most notable differences were observed between the CSC and the SP. Indeed, as observed, the CSC sample stood out for having the lowest anisidine index (1.39 mg/g) and peroxide value (2.4 meq O_2_/kg), indicating minimal oxidation and a fresher fat profile. In contrast, the SP sample showed a much higher anisidine index (18.23 mg/g) and peroxide value (12.5 meq O_2_/kg), reflecting a greater degree of both primary and secondary lipid oxidation. Despite these differences in oxidation status, both samples had similar saponification values, suggesting comparable average fatty acid chain lengths. This clear contrast in fat quality between CSC and SP highlights them as suitable models for studying the impact of different lipid oxidation profiles in subsequent biological assays. Following this detailed characterization, we further narrowed the focus of the present study to Chocolate Sponge Cake (CSC) and Sponge Cake Pastry (SP) for the cell culture experiments. This final selection was guided by their contrasting lipid quality profiles and oxidation status, providing an optimal model system for evaluating the biological effects of food-derived lipids.

### 3.2. Raman and IR

**Figure 3:**
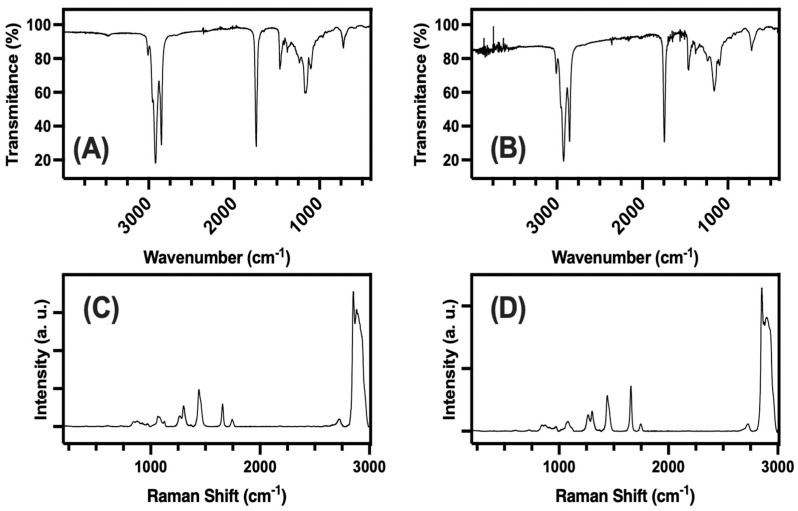
FTIR and Raman spectra of fat extracts from Chocolate Sponge Cake, CSC (**A**,**C**), and Sponge Cake Pastry, SP (**B**,**D**). FTIR spectra (A for CSC, B for SP) show the main absorption bands for C–H and C=O groups typical of triglycerides, while Raman spectra (C for CSC, D for SP) were recorded using a 785 nm excitation laser line, highlighting the strong C–H stretching and other features related to the fatty acid composition in each sample.

The FTIR and Raman spectra for the fat extracts from the selected samples of Chocolate Sponge Cake (CSC, Samples A and C) and Sponge Cake Pastry (SP, Samples B and D) are shown in Figure 3. For the CSC fat (A), the FTIR spectrum showed strong absorption bands at around 2920 cm^−1^ and 2850 cm^−1^, which are typical for the C–H stretching vibrations found in the long chains of fatty acids [48]. There was also a sharp peak near 1740 cm^−1^, which is a clear sign of the ester carbonyl group present in triglycerides, the main molecules in edible fats. The region between 1500 and 1000 cm^−1^ contained several smaller peaks, reflecting the variety of fatty acids and minor components in the chocolate cake’s fat. Also, the band around 966 cm^−1^ served as the fingerprint of the presence of trans fatty acids (TFAs). In fact, this band is characteristic of the C–H out-of-plane deformation vibration found in isolated trans double bonds, which are not present in cis fatty acids [48]. Furthermore, this band had been used widely for rapid and reliable identification and quantification of trans fats in food samples, including fats and oils from baked goods and fast foods. For the SP fat (B), the FTIR spectrum was quite similar, with strong C–H stretching bands and a prominent ester C=O peak at 1740 cm^−1^. The fingerprint region also showed multiple peaks, indicating a mix of fatty acids. Importantly, a small but distinct band is noticeable near 966 cm^−1^ in both samples, which is commonly associated with the presence of trans double bonds in fatty acids, suggesting that trans fatty acids might be present in both types of cake fat extracts, as confirmed through GC-MS [49,50,51].

For its part, the Raman spectrum for CSC fat (C) was dominated by a very strong band in the 2850–2950 cm^−1^ region, very likely due to C–H stretching, and several smaller peaks between 1000 and 1500 cm^−1^, linked to the carbon backbone and side groups of the fatty acids [52]. Similarly, for the SP fat (B), the Raman spectrum for the SP fat (D) displayed the same major C–H stretching band and several smaller peaks in the lower wavenumber region. Despite both types of fat extracts showing similar overall patterns, there were slight differences in the intensity and position of some peaks, which might be related to the specific mix of fats and minor ingredients in each cake type. As expected, the Raman spectra were less sensitive to trans double bonds than FTIR, so the identification of trans fatty acids relied mainly on the FTIR data, particularly the presence of the band at 966 cm^−1^.

### 3.3. Cytotoxicity Assessment

**Figure 4:**
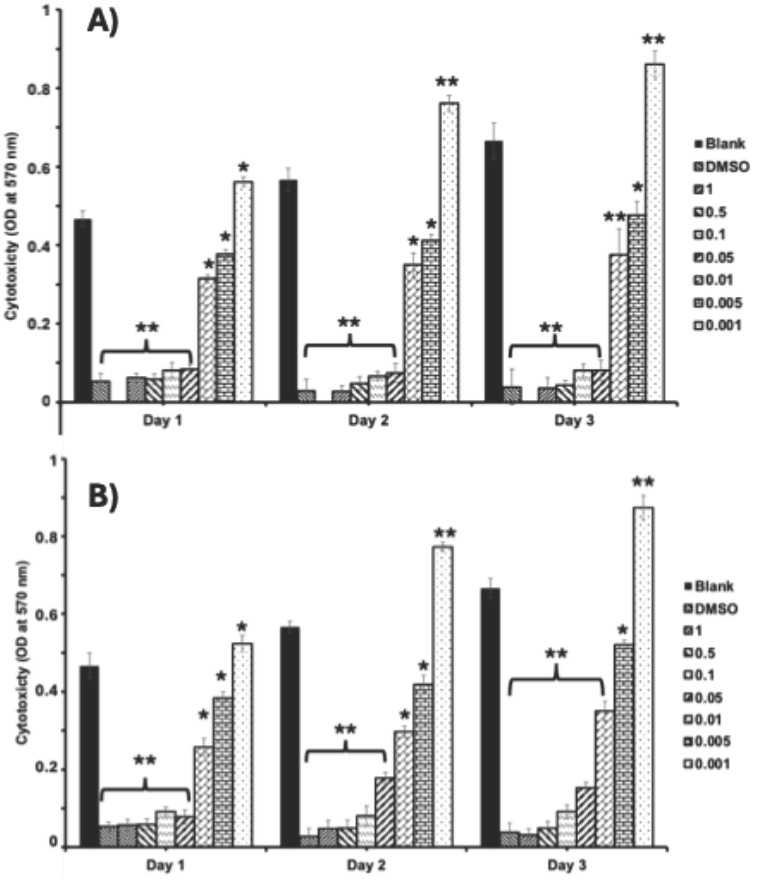
Cytotoxic analysis of TFA samples (SP (**A**) and CSC (**B**)) in MSC cells. Cells were cultured with different concentrations (0.001–1%) of TFA samples for three days. DMSO cells were treated with 1% DMSO and blank cells with culture medium. * *p* < 0.05 and ** *p* < 0.01 denote significant differences.

The cytotoxicity of MSCs was evaluated through optical density at 570 nm of MTT assay over three days for different concentrations of CSC and SP (dose–response curves). In the control group (without treatment), the cell number was increased with respect to the culture time from day 1 to day 3, whereas the cells cultured with DMSO showed significant toxicity as the cell number was decreased starting from day 1 (Figure 4). Comparing the cytotoxicity effects of CSC and SP, both samples exhibited a dose-dependent and time-dependent trend in cytotoxicity, where higher concentrations correlate with increased toxicity over time. However, CSC generally showed lower cytotoxicity in all tested concentrations compared to SP. A comparative assessment of CSC and SP demonstrated a dose-dependent and time-dependent cytotoxic effect. Most importantly, higher concentrations of both compounds correlated with increased toxicity, reducing MSC viability over time. However, CSC consistently exhibited lower cytotoxicity across all tested concentrations compared to SP, suggesting differential cellular responses to these formulations.

Interestingly, while high doses of TFA extracted from CSC and SP (1–0.05%) induced significant toxicity, particularly on days 1 and 2, the lower concentration (0.001%) showed a protective effect, supporting better cell growth than control conditions. Based on the above findings, the potential of optimum-dose TFA extracted from CSC and SP is essential in maintaining MSC viability and suggests a concentration threshold beyond which cytotoxic effects become pronounced.

### 3.4. The Effect of Cell Morphology by TFA

**Figure 5:**
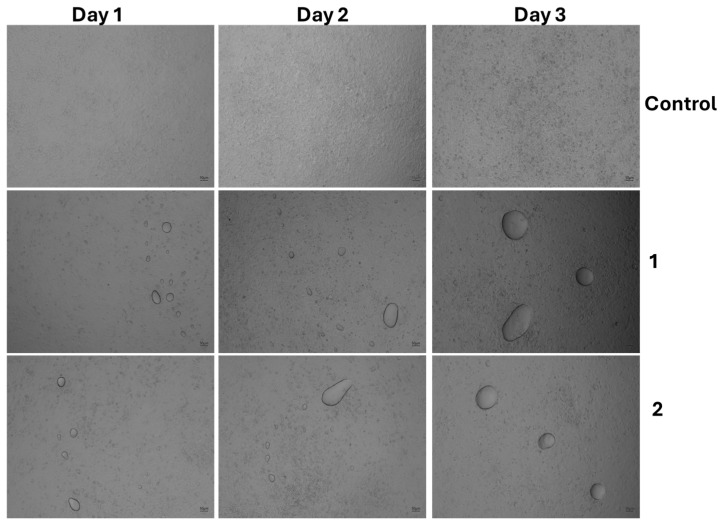

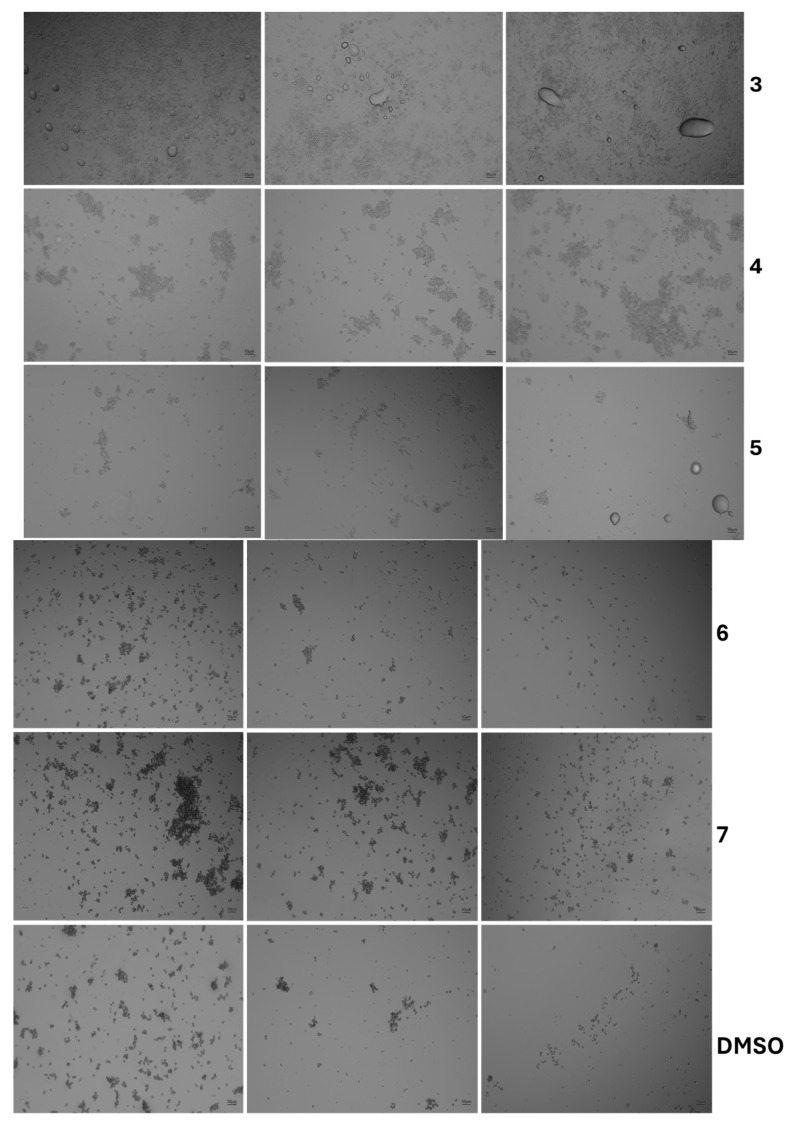
Light microscopic images of MSCs treated with different concentrations of TFA extracted from SP. Cells were cultured with different concentrations (0.001–1%) of TFA for three days. DMSO cells were treated with 1% DMSO and blank cells with culture medium. 1—SP 0.001%, 2—SP 0.005%, 3—SP 0.01%, 4—SP 0.05%, 5—SP 0.1%. 6—SP 0.5%. 7—SP 1%.

**Figure 6:**
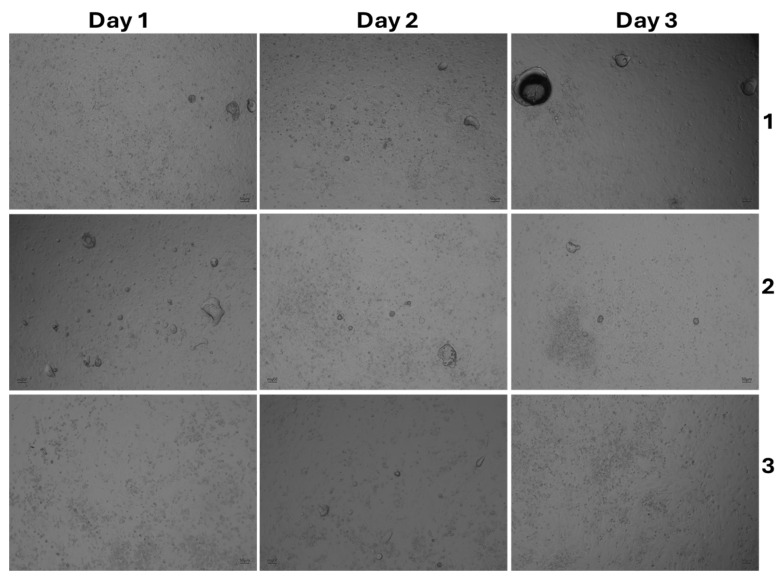

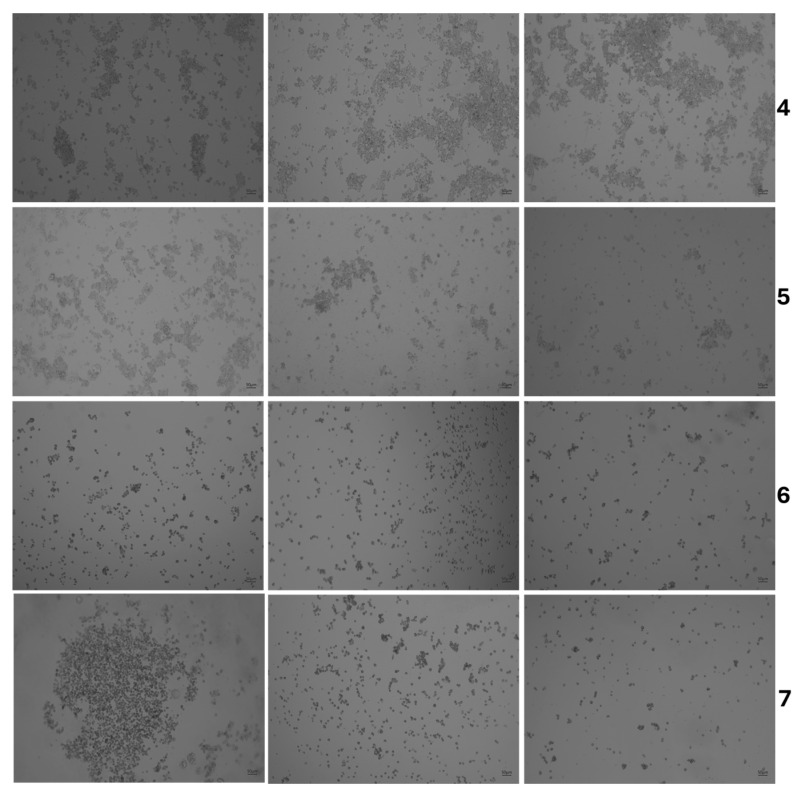
Light microscopic images of MSCs treated with different concentrations of TFA extracted from CSC. Cells were cultured with different concentrations (0.001–1%) of TFA for three days. DMSO cells were treated with 1% DMSO and blank cells with culture medium. 1—CSC 0.001%, 2—CSC 0.005%, 3—CSC 0.01%, 4—CSC 0.05%, 5—CSC 0.1%, 6—CSC 0.5%, and 7—CSC 1%.

**Figure 7:**
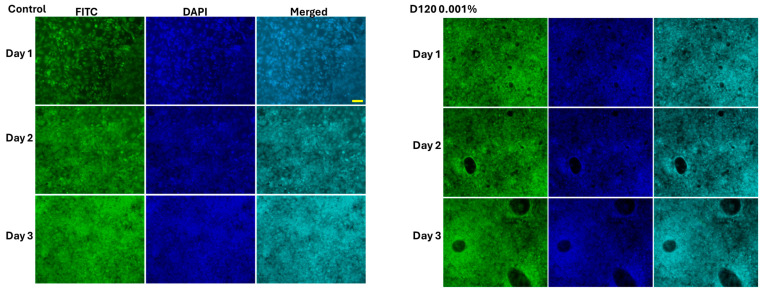

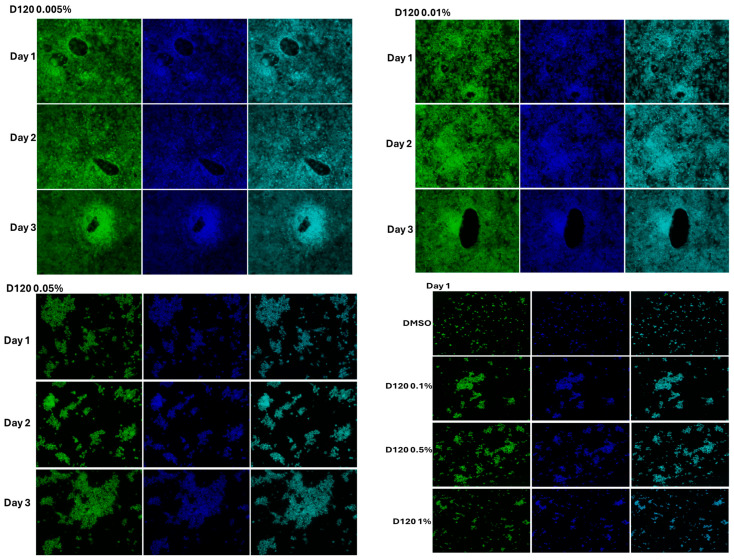
Fluorescent images of MSCs treated with different concentrations of TFA extracted from SP. Cells were cultured with different concentrations (0.001–1%) of TFA for three days. DMSO cells were treated with 1% DMSO and blank cells with culture medium. Green stains—FITC. Blue stains—DAPI.

**Figure 8:**
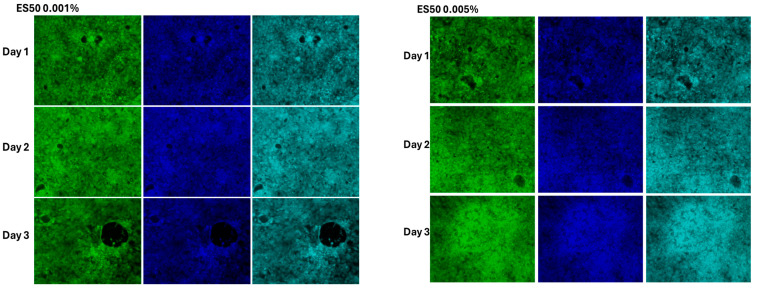

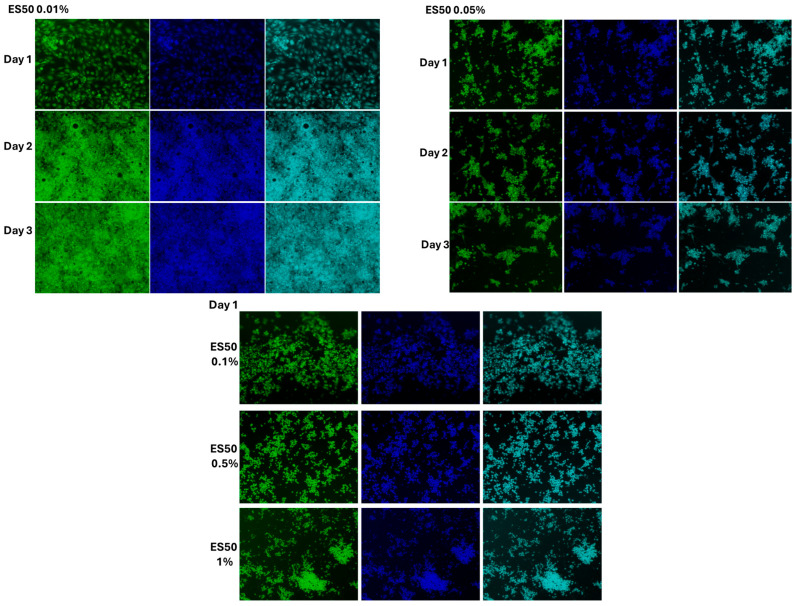
Fluorescent images of MSCs treated with different concentrations of CSC. Cells were cultured with different concentrations (0.001–1%) of TFA samples for three days. DMSO cells were treated with 1% DMSO and blank cells with culture medium. Green stains—FITC. Blue stains—DAPI.

To further confirm the cytotoxic effects of TFAs, MSCs cultured with TFAs were visualized through light (bright-field image) and fluorescence microscopy. Both light and fluorescent (FITC and DAPI) images showed more cellular spreading in the groups treated with less than 0.05% TFAs with regard to the types (Figure 5, Figure 6, Figure 7 and Figure 8). As expected, control cells increased more in day 3 than day 1, whereas the cells cultured with DMSO showed significant toxicity with round cell morphology slightly sticking on the surface, even on day 1. Unfortunately, the cells cultured with both TFAs at higher concentrations (0.05–1%) showed a similar pattern with DMSO, showing their toxic behavior in MSCs. However, cells were partially attached and grown in groups treated with TFAs at a concentration of 0.05% and gradually increased with decreasing concentrations of TFAs. When the concentrations of TFAs reached 0.001%, the MSCs had grown more, and the cells accumulated more in fat globules (shown by the arrow). Although the cytotoxic results showed significant differences between CSC and SP (Figure 5), there was not much difference observed in the microscopic images between them; however, the cells cultured with 0.01%, 0.005%, and 0.001% showed significantly higher cell growth than control groups.

## 4. Discussion

### 4.1. Fat and TFA Content in Pastries and Their Potential Impact on Health

The findings of this study, which detail the fat and fatty acid composition of 11 Spanish pastries, are in line with recent trends observed in the Spanish bakery sector showing a reduction in trans fatty acid (TFA) content compared to earlier decades [7,11,13]. Thus, the present results demonstrate that all analyzed pastries contained TFA levels well below the 2% threshold, with most products exhibiting TFA/total fat ratios under 0.35%. This is consistent with recent Spanish surveys reporting average TFA contents below 0.2 g/100 g of product and a TFA/total fat ratio of less than 2% in the majority of food categories, reflecting successful reformulation efforts by the industry in response to public health recommendations [24]. In contrast, studies from the late 1990s and early 2000s found much higher TFA levels in Spanish bakery products, with mean values of 5–6% and some samples exceeding 15% of total fatty acids, largely due to the use of partially hydrogenated fats [45,53]. Internationally, the TFA values in the samples studied are lower than those reported in Iranian, Turkish, and Malaysian bakery products, where TFA levels can range from 0.5% to over 17% depending on the product and the fat source [54,55]. In terms of total fat, the present results reveal a wide range among Spanish pastries, from less than 2% up to 34.4%, which is comparable to the fat content reported in similar studies from other countries. What distinguishes this study is the detailed sample identification (Table 2) and the integration of compositional data with biological relevance, as we selected two contrasting pastries (Chocolate Sponge Cake (CSC) and Sponge Cake Pastry (SP)) for cell culture experiments to assess the impact of their fat profiles on mesenchymal stem cell function. This approach not only confirms the ongoing success of TFA reduction in Spanish bakery products; it also provides new insights into the potential cellular effects of current fat compositions and their potential effect on health, highlighting the importance of continued monitoring and the need for product-specific nutritional evaluation to inform both public health policy and food manufacturing practices.

Furthermore, a very pronounced difference in lipid quality was observed in the samples studied, particularly in terms of oxidation status. For instance, the SP sample exhibited much higher peroxide and anisidine values compared to CSC, indicating a greater extent of both primary and secondary lipid oxidation. These findings are consistent with those reported in the literature, in which it is claimed that lipid oxidation, measured through indices like peroxide and anisidine values, can vary widely among bakery products and is strongly influenced by both formulation and processing conditions [56,57]. For their part, elevated oxidation indices not only signal a decline in fat quality but are also linked to the formation of off-flavors and reduced nutritional value, which can impact consumer acceptance and product shelf life. The observed differences between CSC and SP underscore the importance of monitoring lipid oxidation in pastries, as these changes can affect both sensory qualities and potential health implications for consumers. These results are in line with the recent literature emphasizing the need for comprehensive assessment of lipid oxidation in foods to ensure quality and safety conditions [56,57,58].

### 4.2. FTIR and Raman

The FTIR and Raman results demonstrated that the main fats extracted from the selected Spanish pastries consist predominantly of triglycerides and long-chain fatty acids, as is typical for this category of baked goods. The strong C–H stretching bands around 2900 cm^−1^ and the sharp ester C=O peak near 1750 cm^−1^ are clear signs of these molecules. When looking more closely at the FTIR spectra, there is a small but noticeable band near 966 cm^−1^ in both samples, which is often linked to the presence of trans fatty acids due to the vibration of trans double bonds. This suggests that both cake types may contain some trans fats, possibly from the fats or margarines used in baking. The Raman spectra, recorded with a 785 nm laser, show the same general pattern for both samples, with strong C–H peaks and several smaller features, but they are less sensitive to trans fats than FTIR. The small differences seen in the fingerprint regions of both FTIR and Raman spectra could be due to slight variations in the types of fats or minor ingredients used in each recipe, such as the presence of butter. These spectroscopic results corroborate not only the similarities in the main fat content of the cakes studied but also subtle differences that could affect texture, flavor, and nutritional value. The results also underline the usefulness of FTIR for identifying, in particular, the 966 cm^−1^ band for detecting trans fatty acids in food samples. Both techniques were thus used as complementary to GC-MS analysis.

### 4.3. Cytotoxicity Assessment

In the present study, TFA samples exhibited dose- and time-dependent effects on MSCs. Cytotoxic effects were observed at concentrations >0.05%, while concentrations of 0.001% promoted cell growth, indicating that the biological impact was strongly dependent on TFA dosage. To support these findings, light microscopic and fluorescence (FITC and DAPI) images also showed the negative effect of TFAs higher than 0.05% in both samples and more cell distribution in cells cultured with lower doses (0.001%) of TFA samples. An earlier study documented the cytotoxic effects of unsaturated fatty acids (UFAs) on tumor cells by Scheim and concluded a concentration threshold between 500 μM and 2 mM necessary for antitumor activity. This highlights the potential of UFAs in selectively targeting malignant cells while raising questions about their mechanism of action in promoting cytotoxicity. Further investigation into the lipid metabolic pathways influenced by UFAs could enhance understanding of their therapeutic implications [59]. Also, Krogager et al. examined the hepatocellular responses to predominant TFA isomers derived from industrial and ruminant sources, namely, elaidic acid and trans-vaccenic acid. Their findings revealed that hepatocellular effects varied significantly depending on the specific isomer, emphasizing the importance of structural differences in lipid metabolism. This distinction suggests that TFAs may differentially influence lipid-mediated stress responses, warranting further examination of their impact on organ function and metabolic disorders [60]. A few other studies have also reported the cytotoxic effects of different TFAs in tumor cells [61,62]. In the present study, the lower cytotoxicity observed with CSC compared to SP appears to be closely associated with its fatty acid composition. Identifying and characterizing the major fatty acids within CSC could provide further insight into their protective or regulatory effects on cell viability. Supporting this perspective, Sarnyai et al. demonstrated that lipotoxicity induced by TFAs, specifically, elaidate and vaccinate, follows distinct metabolic patterns [63]. Their findings revealed that cis and trans C18:1 fatty acid exhibit different metabolic fates, influencing their toxicity in human cells. Notably, TFA incorporation into lipid intermediates, such as ceramides and diacylglycerols, was found to trigger pronounced cellular stress, contributing to apoptosis through ER stress and JNK activation [63]. Rosenthal and Doloresco investigated the effectiveness of different fatty acids as inhibitors in human skin fibroblasts, and the inhibitory effect in skin fibroblast was mild for oleate, elaidate, and linoleate and greater for linoelaidate [6,64]. In a recent study, Hirata et al. reported that industrially produced TFAs (elaidic acid (EA) and linoelaidic acid) were potent promoters of DNA damage-induced apoptosis leading to cardiovascular and neurodegenerative diseases and evaluated underlying mechanisms of TFA in cell death [65]. Also, Hirata et al. have shown that TFAs intensify DNA damage-induced apoptosis in mammalian cells by increasing mitochondrial reactive oxygen species (ROS) production and activating the JNK signaling pathway, thereby amplifying cell death responses [65]. They further demonstrated that TFAs enhance inflammation and apoptosis under cellular stressors, highlighting their distinct pro-apoptotic effects compared to cis isomers and saturated fatty acids. Mechanistically, TFA-induced damage in MSCs occurs through three primary pathways: mitochondrial apoptosis via ROS-triggered JNK-p38 MAPK and caspase activation; inflammatory amplification through TNF-α/TNFR1 and ASK1-p38 signaling; and metabolic disruption involving impaired fatty acid oxidation, endoplasmic reticulum stress, and caspase-3-mediated apoptosis. Understanding how specific fatty acid isomers interact with cellular pathways could pave the way for targeted lipid-based interventions in disease management.

### 4.4. Legal Analysis of Compliance with the Regulatory Framework for Trans Fatty Acids in Spain

The results of this study allow for the identification of both the achievements and the limitations of the Spanish regulatory framework regarding trans fatty acids (TFAs), as well as its degree of alignment with European regulations and the recommendations of the World Health Organization (WHO). Commission Regulation (EU) 2019/649 of 24 April 2019, which amends Regulation (EC) No. 1925/2006, expressly establishes that “the content of trans fats, other than trans fats naturally occurring in fat of animal origin, in food intended for the final consumer and in food intended for supply to retail, shall not exceed 2 g per 100 g of fat” [66]. This provision constitutes the basis of regulatory control in Spain, complemented by Law 17/2011 of 5 July on Food Safety and Nutrition, of which Article 43 requires food business operators to keep records that allow for administrative verification of the TFA content in products [25]. For its part, the WHO recommends that TFA intake should not exceed 1% of total daily energy intake, that is, less than 2.2 g per day for an adult with a standard diet of 2000 kcal, and it urges states to eliminate industrially produced TFAs from the food supply by adopting strict legal limits, robust monitoring systems, and public awareness campaigns [27].

The analysis of Spanish pastries in this study confirms widespread compliance with the legal TFA limit, with levels below 2% of total fat. These findings demonstrate both the effectiveness of reformulation policies and the successful adaptation of the Spanish food industry to regulatory requirements. However, from a legal perspective, this formal compliance is insufficient to guarantee effective protection of public health. The current approach is limited to establishing a maximum per product without incorporating criteria for cumulative intake or considering the frequency or actual amount of consumption, which can lead to exposures above WHO recommendations, especially in vulnerable populations or in contexts of high consumption of ultra-processed products [27,33]. For example, the intake of 1 kg of a product that complies with the legal limit may result in TFA exposure up to nine times higher than the amount recommended by WHO, revealing a significant regulatory gap in terms of public health protection. Additionally, the monitoring and control system in Spain presents structural deficiencies, as it relies largely on industry self-regulation and sporadic inspections, lacking an independent, continuous, and harmonized mechanism for monitoring TFA content in food. This situation contrasts with international guidelines, which advocate for solid and sustained control systems over time, as well as for the standardization of analytical methods for the precise quantification of TFAs, as proposed by WHO and PAHO [27].

On the other hand, the experimental results of this study show that even at concentrations below the legal limit (≥0.05%), TFAs exert cytotoxic effects on mesenchymal stem cells above a threshold, negatively affecting their adhesion, proliferation, and viability. These results strongly suggest that current regulatory limits may not be sufficiently protective and reinforce the need to review legal thresholds in light of the most recent toxicological evidence in accordance with the precautionary principle that governs food safety in the European Union [5]. In view of the above, there is legal justification for the need for regulatory reform towards a more preventive model aligned with WHO recommendations and current scientific evidence. Such reform should include reducing the maximum permitted limit of TFAs in foods below 2%, bringing it closer to the maximum intake objective of 1% of total daily energy, the implementation of an independent and continuous system for monitoring and controlling TFA content in food, mandatory clear and visible labeling of TFA content on all products, as well as the implementation of education and public awareness campaigns on the risks associated with TFAs and the promotion of healthier alternatives in the food industry. These measures would help close the gap between formal regulatory compliance and real protection of public health, moving towards the effective elimination of industrially produced trans fatty acids in Spain and ensuring the consistency of the national legal framework with the global objectives of the WHO.

## 5. Conclusions

This study demonstrated that the trans fatty acid (TFA) content in 11 commonly consumed Spanish pastries is below the 2% threshold established by European and WHO guidelines, reflecting the effectiveness of reformulation policies and regulatory adaptation within the Spanish food industry. However, in vitro experiments revealed that TFA concentrations at or above 0.05% reduced mesenchymal stem cell (MSC) viability and altered cell morphology, suggesting potential cytotoxic effects even at levels currently deemed acceptable. These findings indicate that despite regulatory compliance, frequent or high consumption of such products may still pose health risks, particularly for vulnerable populations. The newly developed extraction and analysis method proved reliable and may be useful for future research and routine food safety monitoring. Despite the fact that the Spanish regulatory framework has made significant progress, limitations remain, particularly regarding the product-based approach and the lack of continuous monitoring. Thus, these results support the need for ongoing surveillance, potential revision of legal limits, improved consumer education, and further in vivo and clinical studies to fully assess the health implications of TFAs and advance towards their elimination, in line with WHO objectives.

## Figures and Tables

**Table 1:** Product names and codes for Spanish pastries analyzed.

Product Name	Code
Cookie-01	C01
Cookie-02	C02
Cookie-03	C03
Sponge Cake-01	SC01
Sponge Cake-02	SC02
Sponge Cake-03	SC03
Sponge Cake-04	SC04
Chocolate Palmier	CP
Sponge Cake Pastry	SP
Chocolate Sponge Cake	CSC
Valencia Sponge Cake	VSC

**Table 2:** Fatty acid composition and trans fatty acid content (%) of Spanish pastries analyzed through GC-MS.

Fatty Acid	C01	C02	C03	SC01	SC02	SC03	SC04	CP	SP	CSC	VSC
C-12:0 (Lauric)	0.01 ± 0.01	0.12 ± 0.01	0.37 ± 0.02	0.04 ± 0.01	0.03 ± 0.01	0.01 ± 0.01	0.02 ± 0.01	17.19 ± 0.5	0.18 ± 0.01	15.68 ± 0.4	0.01 ± 0.01
C-14:0 (Myristic)	0.06 ± 0.01	0.13 ± 0.01	0.91 ± 0.03	0.33 ± 0.02	0.27 ± 0.01	0.30 ± 0.01	0.27 ± 0.01	6.56 ± 0.2	0.67 ± 0.02	6.07 ± 0.2	0.08 ± 0.01
C-16:0 (Palmitic)	5.15 ± 0.10	5.90 ± 0.10	39.89 ± 0.5	22.65 ± 0.3	17.35 ± 0.2	22.22 ± 0.3	17.97 ± 0.2	28.91 ± 0.4	8.56 ± 0.10	8.74 ± 0.10	6.80 ± 0.10
C-16:1 (Palmitoleic)	0.17 ± 0.01	0.16 ± 0.01	0.19 ± 0.01	2.41 ± 0.05	2.11 ± 0.05	3.41 ± 0.08	2.43 ± 0.05	0.10 ± 0.01	0.42 ± 0.01	0.19 ± 0.01	0.24 ± 0.01
C-17:0 (Margaric)	0.04 ± 0.01	0.04 ± 0.01	0.10 ± 0.01	0.15 ± 0.01	0.13 ± 0.01	0.18 ± 0.01	0.13 ± 0.01	0.08 ± 0.01	0.07 ± 0.01	0.04 ± 0.01	0.04 ± 0.01
C-17:1 (Margaroleic)	0.05 ± 0.01	0.05 ± 0.01	0.03 ± 0.01	0.10 ± 0.01	0.08 ± 0.01	0.08 ± 0.01	0.12 ± 0.01	0.02 ± 0.01	0.05 ± 0.01	0.02 ± 0.01	0.04 ± 0.01
C-18:0 (Stearic)	2.81 ± 0.05	2.99 ± 0.05	4.54 ± 0.08	5.71 ± 0.10	4.00 ± 0.08	5.11 ± 0.10	4.06 ± 0.08	10.15 ± 0.20	4.10 ± 0.05	11.32 ± 0.20	3.69 ± 0.05
C-18:1 (Oleic)	77.80 ± 1.0	75.60 ± 1.0	39.80 ± 0.5	44.41 ± 0.5	48.57 ± 0.5	39.42 ± 0.5	48.43 ± 0.5	22.43 ± 0.5	32.19 ± 0.5	20.90 ± 0.5	32.45 ± 0.5
C-18:2 (Linoleic)	11.85 ± 0.20	12.93 ± 0.20	12.86 ± 0.20	21.47 ± 0.30	23.20 ± 0.30	27.19 ± 0.30	22.43 ± 0.30	11.81 ± 0.20	52.03 ± 0.50	34.09 ± 0.50	55.04 ± 0.50
C-20:0 (Arachidic)	0.27 ± 0.01	0.28 ± 0.01	0.39 ± 0.01	0.18 ± 0.01	0.28 ± 0.01	0.05 ± 0.01	0.26 ± 0.01	0.32 ± 0.01	0.26 ± 0.01	0.29 ± 0.01	0.26 ± 0.01
C-18:3 (Linolenic)	0.36 ± 0.01	0.37 ± 0.01	0.53 ± 0.01	1.94 ± 0.05	3.06 ± 0.08	1.65 ± 0.05	3.10 ± 0.08	0.15 ± 0.01	0.13 ± 0.01	0.13 ± 0.01	0.10 ± 0.01
C-20:1 (Eicosenoic)	0.28 ± 0.01	0.28 ± 0.01	0.19 ± 0.01	0.43 ± 0.01	0.61 ± 0.01	0.33 ± 0.01	0.57 ± 0.01	0.10 ± 0.01	0.17 ± 0.01	0.11 ± 0.01	0.17 ± 0.01
C-22:0 (Behenic)	0.85 ± 0.01	0.86 ± 0.01	0.10 ± 0.01	0.09 ± 0.01	0.12 ± 0.01	0.03 ± 0.01	0.10 ± 0.01	0.17 ± 0.01	0.75 ± 0.01	0.51 ± 0.01	0.80 ± 0.01
C-22:1 (Erucic)	0.01 ± 0.01	0.01 ± 0.01	0.01 ± 0.01	0.04 ± 0.01	0.12 ± 0.01	0.01 ± 0.01	0.07 ± 0.01	0.01 ± 0.01	0.01 ± 0.01	0.01 ± 0.01	0.01 ± 0.01
C-24:0 (Lignoceric)	0.32 ± 0.01	0.31 ± 0.01	0.08 ± 0.01	0.04 ± 0.01	0.08 ± 0.01	0.03 ± 0.01	0.05 ± 0.01	0.10 ± 0.01	0.27 ± 0.01	0.21 ± 0.01	0.29 ± 0.01
T. Oleic (t-C18:1)	0.04 ± 0.01	0.04 ± 0.01	0.07 ± 0.01	0.13 ± 0.01	0.13 ± 0.01	0.15 ± 0.01	0.14 ± 0.01	0.06 ± 0.01	0.14 ± 0.01	0.03 ± 0.01	0.05 ± 0.01
T. Linoleic + Linolenic	0.05 ± 0.01	0.06 ± 0.01	0.28 ± 0.01	0.12 ± 0.01	0.17 ± 0.01	0.14 ± 0.01	0.14 ± 0.01	0.12 ± 0.01	0.09 ± 0.01	0.08 ± 0.01	0.14 ± 0.01
Total Fats	9.04 ± 0.10	9.84 ± 0.10	8.09 ± 0.10	3.81 ± 0.10	2.83 ± 0.10	1.81 ± 0.10	2.76 ± 0.10	34.4 ± 0.5	27.9 ± 0.5	25.6 ± 0.5	25.3 ± 0.5
Total Trans Fatty Acids	0.09 ± 0.01	0.10 ± 0.01	0.35 ± 0.01	0.25 ± 0.01	0.30 ± 0.01	0.29 ± 0.01	0.28 ± 0.01	0.18 ± 0.01	0.24 ± 0.01	0.11 ± 0.01	0.20 ± 0.01

Values represent mean ± standard deviation (SD) from *n* = 3 independent replicates. SDs reflect analytical precision of GC-MS quantification methods under optimized conditions.

**Table 3:** Quality and composition indices of fat extracts from selected Spanish pastries.

Sample	Acidity Index (mg KOH/g)	Anisidine Index (mg/g)	Peroxide Value (meq O_2_/kg)	Iodine Value (g I_2_/100 g)	Saponification Value (mg KOH/g)
CP	1.94 ± 0.06	18.23 ± 0.25	12.5 ± 0.3	80 ± 2	190 ± 2
CSC	1.71 ± 0.05	1.39 ± 0.05	2.4 ± 0.08	66 ± 2	187 ± 2
SP	1.49 ± 0.05	15.68 ± 0.20	10.2 ± 0.3	78 ± 2	194 ± 2
VSC	0.79 ± 0.03	11.72 ± 0.15	6.8 ± 0.2	84 ± 2	197 ± 2

## Data Availability

The original contributions presented in the study are included in the article; further inquiries can be directed to the corresponding authors.
